# Margin selection to compensate for loss of target dose coverage due to target motion during external‐beam radiation therapy of the lung

**DOI:** 10.1120/jacmp.v16i1.5089

**Published:** 2015-01-08

**Authors:** W Kyle Foster, Ernest Osei, Rob Barnett

**Affiliations:** ^1^ Medical Physics Department Grand River Regional Cancer Centre Kitchener ON; ^2^ Department of Physics and Astronomy University of Waterloo Waterloo ON; ^3^ Physics and Engineering Department London Regional Cancer Program London ON Canada

**Keywords:** treatment margin, convolution, target motion, dose coverage

## Abstract

The aim of this study is to provide guidelines for the selection of external‐beam radiation therapy target margins to compensate for target motion in the lung during treatment planning. A convolution model was employed to predict the effect of target motion on the delivered dose distribution. The accuracy of the model was confirmed with radiochromic film measurements in both static and dynamic phantom modes. 502 unique patient breathing traces were recorded and used to simulate the effect of target motion on a dose distribution. A 1D probability density function (PDF) representing the position of the target throughout the breathing cycle was generated from each breathing trace obtained during 4D CT. Changes in the target $D_{95}$ (the minimum dose received by 95% of the treatment target) due to target motion were analyzed and shown to correlate with the standard deviation of the PDF. Furthermore, the amount of target $D_{95}$ recovered per millimeter of increased field width was also shown to correlate with the standard deviation of the PDF. The sensitivity of changes in dose coverage with respect to target size was also determined. Margin selection recommendations that can be used to compensate for loss of target $D_{95}$ were generated based on the simulation results. These results are discussed in the context of clinical plans. We conclude that, for PDF standard deviations less than 0.4 cm with target sizes greater than 5 cm, little or no additional margins are required. Targets which are smaller than 5 cm with PDF standard deviations larger than 0.4 cm are most susceptible to loss of coverage. The largest additional required margin in this study was determined to be 8 mm.

PACS numbers: 87.53.Bn, 87.53.Kn, 87.55.D‐, 87.55.Gh

## I. INTRODUCTION

The management of target motion during external‐beam radiation therapy (EBRT) is crucial for ensuring agreement between prescribed and delivered dose to the patient and, hence, successful treatment outcomes. Organ motion that occurs during the delivery of radiation therapy is referred to as intrafraction motion. Intrafraction motion can originate from a variety of sources such as cardiac motion, gastrointestinal peristalses or respiratory motion.^(1)^ The works of other authors have described the motion of targets in the lung due to respiration.^2^, ^3^, ^4^ It is generally noted that target motion in the lung is largest in the superior–inferior (SI) direction, although each patient presents with unique motion. It has also been shown that target motion resulting from patient respiration can cause the displacement of lesions in the lung by up to 2 cm in some cases.^(5)^

A report by the American Association of Physicists in Medicine (AAPM) Task Group TG‐76^(1)^ highlights several different methods for managing respiratory motion. These include motion encompassing techniques (margin and target volume definitions), various breath‐hold techniques, gated treatment delivery, and target tracking methods. Each of these methods presents tradeoffs between dose conformality, technical feasibility, demand on clinical resources, and patient condition. The least technically demanding (and therefore most widely used) of these approaches is to define treatment volumes that account for the expected motion of the target. Within this approach to motion management there are several different methods which can be used to define treatment volumes. Widely accepted motion encompassing techniques include margin expansion,^6^, ^7^, ^8^ the use of an internal target volume (ITV) determined by the range of target locations,^9^, ^10^ and probabilistic approaches to volume definition.^(11)^ Because the expansion of treatment volumes is accompanied by an increase in normal tissue complication probability (NTCP), a balance must be struck between improving target dose coverage with larger treatment volumes and potential complications to healthy tissues.

The increased availability of 4D CT scans has increased the viability of patient‐specific approaches. 4D CT datasets provide clinicians with information regarding the location of the target during various phases of a given patient's respiratory cycle,^(12)^ as well as information regarding the patient's breathing pattern.^(10)^ One calculation model used to predict the effect of target motion on the delivered dose distribution is the convolution model originally proposed by Leong.^(13)^ This model takes a planned static dose distribution and a probability density function (PDF) describing the target location over time as its inputs. The model predicts the delivered dose distribution by performing a mathematical convolution of the inputs. The result of this convolution is a dose distribution which has been ‘blurred’ by the motion of the target, thereby reducing target dose coverage and increasing dose to organs at risk. As reported by other authors,^(14)^ this blurring is most predominant in regions of the dose distribution with sharp dose gradients. Since the sharp dose gradients are typically associated with field edges, selection of appropriate target margins becomes a crucial step in ensuring adequate target coverage.

In searching for the appropriate balance it becomes clear that patient‐specific approaches to target volume definition are necessary because of the wide variation in patient anatomy, disease manifestation, and breathing patterns. Furthermore, some lung cancer patients present with highly irregular breathing patterns, characterized by large inter‐ or intrafraction changes to target motion amplitude or cycle frequency. These patients present unique challenges with respect to modeling and predicting the target position throughout the breathing cycle and will be less amenable to a class solution. However, by developing margin selection guidelines that can be implemented by any radiation treatment center, the benefits of a patient‐specific approach can be easily attained. Therefore, the aims of this work are as follows: 1) to use a convolution model to provide estimates of the loss of target dose coverage which can be expected as a result the dose blurring effect of a given respiratory motion pattern; and 2) to provide guidelines for the additional margin required to compensate for the predicted loss of target dose coverage for a given patient.

## II. MATERIALS AND METHODS

### A. Convolution model

The effect of intrafraction target motion on the delivered dose distribution is predicted by extending the convolution model proposed by Leong.^(13)^ While this method was originally used to model the effect of interfraction motion, it can also be applied to intrafraction motion with some modification. Lujan et al.^(14)^ also used this model to analyze breathing motion. That work was done in the context of fluoroscopic imaging, as opposed to the modern context of 4D CT that is used in this work.

The model predicts the delivered (or blurred) dose distribution, $D_{b}$, by performing a convolution using the static (or planned) dose distribution, $D_{s}$, and a probability distribution function (PDF) describing the varying position of the target during treatment delivery. Thus

(1) $$D_{b}(\vec{r})=D_{s}(\vec{r})⊗PDF(\vec{r})$$

It should be noted that $D_{s}$ refers to the dose distribution expected to be delivered to a static target. The model makes the assumption that this static distribution remains constant through the entire treatment. The model assumptions are reviewed in more detail in the Discussion section.

A modification to this model was proposed by Jiang et al.^(15)^ to explicitly identify the gradient of the static dose distribution. Given that the regions of the dose distribution with the steepest gradients are most susceptible to target motion, this approach highlights regions of the treatment plan that require additional care with respect to compensation for target motion.

(2) $$D_{b}(\vec{r})=\int \nabla D_{s}(\vec{r})⊗PDF(\vec{r})d\vec{r}$$

Furthermore, it was shown by Jiang et al.^(15)^ that the gradient of the PDF could equivalently be taken instead of the gradient of the static distribution, $D_{s}$.

(3) $$D_{b}(\vec{r})=\int D_{s}(\vec{r})⊗\nabla PDF(\vec{r})d\vec{r}$$

Equation (3) highlights the features of the PDF which have the predominant effect on the delivered dose distribution. This work is primarily concerned with the principal superior–inferior (SI) motion of lung cancers, and so the results presented are in one dimension. However, these methods can be extended to two and three dimensions. In the one‐dimensional case, Eq. (3) reduces to:

(4) $$D_{b}(x)=\int D_{s}(x)⊗\frac{d}{dx}PDF(x)dx$$

### B. Patient breathing traces and PDFs

The PDFs used for this work were generated from patient breathing traces which were recorded at the London Regional Cancer Program (London, Ontario) using the Varian RPM system (Varian Medical Systems, Inc., Palo Alto, CA). The breathing traces were acquired during 4D CT simulation and were originally used for 4D CT image reconstruction. A histogram was generated from each breathing trace recorded during the 4D CT ‘beam on’ time using MATLAB (The MathWorks, Inc., Natick, MA). A curve was then fitted to the histogram and the resulting function was normalized such that the area under the curve was unity to arrive at the PDF. The origin of the PDF coordinate system was placed at the mean location of the recorded positions, as suggested by other authors.^(8)^ Breathing patterns from a total of 502 unique patients were used in the simulation study. No consideration was given to the disease sites, although the majority of cases (∼80%) were lung cancer patients. Abdominal disease sites comprised most other cases.

For the purposes of this analysis, the breathing trace amplitudes, as recorded by the Varian RPM system, were assumed to be equal to the target motion amplitude within the patient. This was done to ensure a wide range of motion amplitudes were considered. Although the amplitude of target motion within the patient is not equal to the amplitude of the marker block motion in general, the breathing trace amplitude can be renormalized^(7)^ to the target motion amplitude as seen on 4D CT. A breathing trace renormalized in this way is representative of one‐dimensional motion of the target during breathing. It is recognized that using an external surrogate for target motion does present problems in terms of reproducibility from day to day. However, in this study the surrogate is only used initially to generate the PDF. As long as the patient is able to reproduce their breathing pattern in terms of a consistent standard deviation, the recommended margins will remain valid. In practice, this means the patient must reproduce the amplitude and frequency of breaths to a reasonable degree; they do no need to be exactly the same as originally measured.

### C. Film measurements

#### C.1 Film calibration and handling

GAFCHROMIC EBT2 film (ISP, Wayne, NJ) of the same batch (lot # A02181104) was calibrated and handled according to the procedures laid out in AAPM TG‐55^(16)^ and the recommendations of the manufacturer. Film calibration was performed in a Solid Water phantom set up in reference geometry, such that 1 MU delivered 1 cGy to the center of the film. All the films were digitized with an EPSON 10000XL flatbed scanner (US Epson, Long Beach, CA) 22 hrs after irradiation. This time frame was selected to allow for the film to develop and to provide consistency in the handling procedure. Calibration and analysis of the images was performed using the RIT v1.2 software package (Radiological Imaging Technology, Inc., Colorado Springs, CO). RIT recommends using 13 dose points as part of the calibration curve. For this study with maximum dose of 200 cGy, the dose values used for calibration were: 300, 240, 200, 160, 120, 90, 70, 50, 40, 30, 20, 10, and 0 cGy. The calibration curve was generated from the film response to these doses and fit using a cubic spline. Careful handling and processing of the films used for calibration and measurement allows for absolute dose measurements to be acquired with a relative uncertainty of approximately ±6%.^(17)^

#### C.2 Film measurements in static phantom

In order to verify the delivery of the dose distribution predicted by Eclipse v10.1 treatment planning software (TPS) (Varian Medical Systems) using the AAA dose calculation algorithm, GAFCHROMIC EBT2 film measurements were taken in a CIRS Dynamic Thorax Phantom, Model 008 (Computerized Imaging Reference Systems, Inc., Norfolk, VA). This phantom has multiple inserts, each of which accepts a different dosimeter. For this work the film insert was used. The phantom can also be programmed to replicate one‐dimensional target motion (dynamic mode) during irradiation, or it can be used in a static mode with no motion. Film measurements were taken in the thorax phantom in both static and dynamic modes for the verification of the delivered dose distributions.

A CT simulation of the static phantom was acquired using a typical departmental protocol with the lung‐equivalent midplane film insert in place. A simple, open MLC, three‐field plan was generated (Table 1) with the Eclipse TPS. The beam isocenters were placed at the center of the film insert within the phantom. The dose rate was 400 MU/min for all beams and all couch angles were set to 0°. Multiple film measurements of the dose delivered to the static phantom were acquired. The absolute dose profiles taken from these films were compared to the dose profiles calculated by Eclipse at the same location inside the phantom. Although the film insert used for this study does not include a target for dose buildup, this experimental setup provides a representative case. The effect of tissue inhomogeneities on the convolution model was analyzed by Craig et al.^(18)^ and shown to be of little consequence to this type of work.

**Table 1:** Parameters of the beam arrangement used to generate the static dose distributions used in the simulation study. The coordinates system used is the IEC convention

	*Beam 1*	*Beam 2*	*Beam 3*
Gantry Angle (deg)	315	0	45
(X, Y) Jaw settings (cm)	(3.7, 3.7)	(3.2, 3.7)	(3.6, 3.7)
MU	86.6	92.5	93.9

#### C.3 Film measurements in dynamic phantom

Film measurements were also taken with the phantom in the dynamic mode. This was achieved by programming the one‐dimensional motion of the phantom insert to replicate the motion of selected patient breathing traces. Several different patient breathing traces were used, some with regular repetitive breathing and others with highly irregular breathing patterns. With the film insert in motion within the phantom, the same three‐field treatment plan used for the static case was delivered to the target. The absolute dose profiles from the films acquired in the dynamic mode were compared to the dose profile predictions made by the convolution model discussed above in Materials & Methods section A.

### D. Simulation study

#### D.1 Simulation study — Loss of target dose coverage due to target motion

The effect of motion on the delivered dose distribution as predicted by the convolution model was simulated for each of the 502 patient breathing traces. An in‐house MATLAB program was used to perform the convolutions according to Eq. (4), and analyze the dose coverage offered by the resulting profiles to the spherical (37 mm diameter) planning target volume (PTV). The coverage was described by using the $D_{95}$ metric (the minimum dose received by 95% of the PTV). Furthermore, several different static dose profiles were created by incrementally modifying the beam width of the treatment plan using the jaw settings only. For the purposes of this work, the changes in beam width were defined relative to the width of the original PTV defined on the static phantom (37 mm). The additional beam width was added to each beam and ranged from 0 mm to 13 mm in 1 mm increments. The central axis of the beams remained targeted to the center of the PTV. Therefore, an additional 1 mm of beam width results in an additional margin of 0.5 mm on each side (superior and inferior) of the target. Changes to the beam width were only made in the same direction as the target motion (SI). This aspect of the simulation study consisted of the analysis of 7,028 dose profiles resulting from each breathing trace being convolved with each of the 14 static dose profiles. The change in dose coverage to the target was then compared with various statistics of the PDF in order to find trends that could be used to predict loss of coverage based on patient breathing and guide the selection of margins required to compensate for the loss of coverage.

#### D.2 Simulation study — the effect of target size on loss of dose coverage

The role of target size on loss of dose coverage was analyzed by convolving dose profiles of varying full width at half max (FWHM) values against the full set of PDFs. Twelve dose profiles with FWHM values ranging from 2.6 cm to 10.1 cm were used for this aspect of the simulation study. The shape of these profiles is consistent, with a flat dose region in the center and sharp penumbral regions typical of simple plans at the edges. The 80%–20% penumbral region spanned 8 mm in each case. By varying the FHWM of the profiles while maintaining the shape of the penumbral regions, the effect of target size on loss of dose coverage due to target motion can be analyzed in isolation.

#### D.3 Simulation study — clinical treatment plans

Three clinical treatment plans were also selected for motion simulation: a three‐field, a four‐field and an arc plan, all of which were used to treat patients at our clinic. The dose profiles from these plans were convolved with the same set of breathing traces. The clinical dose profiles and target definitions that were originally used in these plans were kept the same for the purposes of this simulation. The resulting loss of target coverage in the respective PTVs was recorded in order to assess the effect of motion in clinically relevant situations.

## III. RESULTS

### A. Static mode film measurements

**Figure 1:**
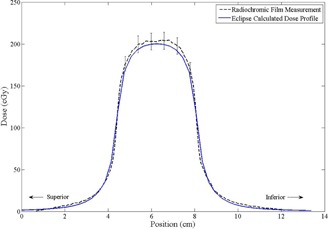
Static dose profile measurement vs. calculated prediction from Eclipse. The error bars represent the margin of error for good handling (±6%). The agreement is good, with 96.1% of dose points meeting the 3%/3 mm (calculated in 1D) criteria commonly used for quality assurance.

The film measurements taken in the static mode were used to demonstrate the repeatability and accuracy of the film handling and measurement procedures. These results also serve to verify the calculations made by the Eclipse TPS environment. The dose profiles resulting from the static phantom film measurements are shown in Fig. 1. The absolute dose profiles have good agreement with the predictions made by Eclipse. In this case, 96.1% of the measured dose points are within 3% or 3 mm of the corresponding local dose points in the Eclipse prediction. These criteria are commonly used when assessing the level of agreement between a measured and calculated dose plane in the context of a gamma evaluation,^(19)^ and are similar to the recommendations of Van Dyk et al.^(20)^ for a quality assurance threshold.

### B. Dynamic mode film measurements

The film measurements taken with the phantom in the dynamic setting were used to verify the ability of the convolution model to predict the effect of intrafraction motion on the delivered dose distribution. Film measurements were taken with the phantom in the dynamic mode and mimicking a one‐dimensional, rigid motion of several different patient breathing traces. 2, 3 present the results of two such dynamic phantom film measurements. The selected traces were used to demonstrate that the convolution model makes valid predictions for a wide range of breathing patterns, including regular and irregular breathers.

**Figure 2:**
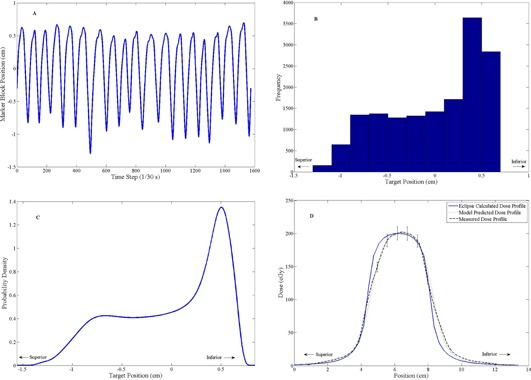
A regular breathing trace (a) used to program the dynamic thorax phantom. The associated target position histogram (b). The PDF (c) generated from the histogram. Dynamic mode dose profile measurements (d) demonstrating the applicability of the convolution model for intrafraction motion. The predictions of the model were in good agreement with the measured dose, with 98.9% of dose points meeting the 3%/3 mm criteria (calculated in 1D). The error bars represent the ±6% dose error.

**Figure 3:**
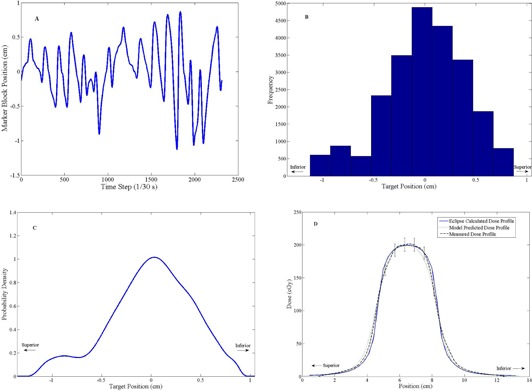
An irregular breathing trace (a) used to program the dynamic thorax phantom. The associated target position histogram (b). The PDF (c) generated from the histogram. Dynamic mode dose profile measurements (d) demonstrating the applicability of the convolution model for intrafraction motion. The predictions of the model were in good agreement with the measured dose (within ±6%), with 98.1% of dose points meeting the 3%/3 mm criteria.

In the case of the regular breathing pattern (Fig. 2), 98.9% of the measured dose points passed the 3%/3 mm criteria (local dose difference), while the measurement of the irregular breathing pattern (Fig. 3) dose distribution passed on 98.1% of dose points.

#### C.1 Simulation study — loss of target coverage

**Figure 4:**
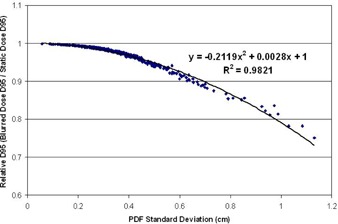
A plot of the relative target $D_{95}$ (blurred dose $D_{95}$ / static dose $D_{95}$) vs. the breathing trace standard deviation for 502 unique breathing traces. The $D_{95}$ was calculated on the simulated 1D dose profiles. The loss of target $D_{95}$ can be predicted based on the standard deviation of the patient's specific breathing pattern PDF. Once the loss of coverage is determined, the required additional margin can be assessed.

The simulation study was used to look for trends in target dose coverage that could be used to guide treatment planning and margin selection decisions. In order to make margin selection recommendations, it is useful to know the loss of target coverage which is expected on case‐by‐case basis. This simulation study revealed that the loss of target $D_{95}$ is predictable, based on the patient's specific PDF standard deviation. Figure 4 demonstrates the relationship between the expected loss of target $D_{95}$ (in terms of the ratio of the static and blurred $D_{95}$) and the standard deviation of the breathing pattern. These results are in line with data presented by other authors^7^, ^8^ regarding the predictive power of the PDF standard deviation with regard to the target dose coverage.

**Figure 5:**
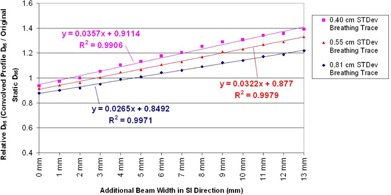
A plot of the linear relation between the relative $D_{95}$ coverage (blurred dose $D_{95}$ / static dose $D_{95}$) and the beam width in the direction of motion. A similar curve was generated for each of the 502 available breathing patterns, in order to establish the trend across a wide range of patient breathing patterns.

The next assessment was to determine target $D_{95}$ can be recovered by incrementally increasing the beam width. By analyzing the dose coverage predicted by the convolution model for several beam widths and all the available breathing patterns, it was established that the rate of change of target $D_{95}$ is linear with changes in the planned beam width. Figure 5 shows the target dose coverage predicted by the convolution model at varying beam widths, for three selected breathing patterns. In each case, a linear fit to the data shows a strong correlation between the beam width and the resulting target coverage. This linear relation can be used to guide the selection of treatment margins for a given patient. A plot of the coverage data and linear fit were generated for each of the 502 breathing patterns available. The statistics summarizing the strength of these fits are presented in Table 2. The average $R^{2}$ value of these fits was 0.9901 with a standard deviation of 0.0067, indicating that the linear trend in dose coverage versus beam width is present across all breathing patterns.

**Table 2:** Statistics summarizing the strength of the linear fits for all breathing patterns

*Statistic*	*Number of Traces*	*Average* $R^{2}$ *of Linear Fit*	*Standard Deviation of* $R^{2}$ *Values*	*Minimum* $R^{2}$ *Value*	*Maximum* $R^{2}$ *Value*
Value	502	0.9901	0.0067	0.9668	0.9995

**Figure 6:**
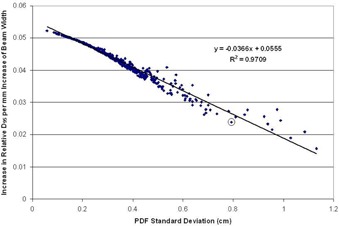
A plot of the increase in target $D_{95}$ per mm of beam width vs. the standard deviation of the PDF. This plot indicates that the standard deviation of the breathing PDF is a strong predictor of the fit slopes demonstrated in Fig. 5. For example, this plot shows that an increase of 1 mm of beam width will contribute an additional 4 percentage points of relative $D_{95}$ to the target in the case of a breathing pattern with a standard deviation of 0.4 cm. The circled point is indicated for later discussion.

Given that the trend in coverage is linear, the slope of the fit defines the rate of change in target dose coverage (in terms of $D_{95}$) per millimeter change in field size. However, the slope of each fit is dependent on the specific patient breathing pattern. Fortunately, the standard deviation of a given breathing PDF is also a strong predictor of the slope of the ft. Figure 6 is a plot of the fit slope versus the standard deviation of the breathing PDF. By using the loss of target $D_{95}$ predicted by the PDF standard deviation and the rate of target $D_{95}$ recovered per millimeter of increased field size, margin recommendations can be made.

#### C.2 Simulation study — the effect of target size on loss of dose coverage

It is necessary to consider the role of target size when trying to compensate for motion and deliver a specific $D_{95}$ to the target. As the FWHM of the profiles increases, the relative contribution of the penumbral region of the dose profile decreases. Since the penumbral region of the profile is predominantly affected by the convolution, smaller targets will be more susceptible to loss of dose coverage due to motion as compared to larger targets.

**Figure 7:**
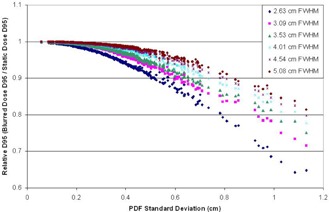
A plot of the relative $D_{95}$ vs. the PDF standard deviation for phantom plans of varying widths. The penumbral region of each plan was the same, with the 80%–20% region spanning 8 mm in each case. A clear trend can be seen with smaller targets being more susceptible to loss of dose coverage as compared to larger targets. The relative $D_{95}$ data for larger targets starts becoming coincident with the data for the 5.1 cm FHWM profile and were omitted from the figure.

The result of this simulation study is summarized in Fig. 7. As the FHWM of the various profiles increases, the given profile becomes less sensitive to motion in terms of loss of $D_{95}$. For example, the target profile with a FHWM of 2.6 cm loses approximately 7% of its original $D_{95}$ when faced with target motion of 0.4 cm standard deviation. The same level of motion causes a loss of approximately 1.5% of the original $D_{95}$ to a target profile with a FHWM of 5.1 cm. As the target FWHM increases beyond 5.1 cm, the changes in sensitivity to motion become small. This is due to the smaller relative contribution of the penumbral regions to wider profiles. As a result, the $D_{95}$ data for targets with FWHM greater than 5.1 cm begin to overlap with the data for the 5.1 cm target and were, therefore, omitted from Fig. 7.

#### C.3 Simulation study — clinical treatment plans

**Figure 8:**
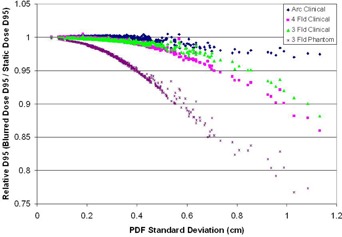
A plot of the relative $D_{95}$ vs. the PDF standard deviation for three clinical plans with the phantom data for comparison. The clinical plans with typical margins selected are robust in the face of target motion up to a PDF standard deviation of approximately 4 mm. For target motion beyond this level, additional margins would be required to maintain target $D_{95}$.

**Figure 9:**
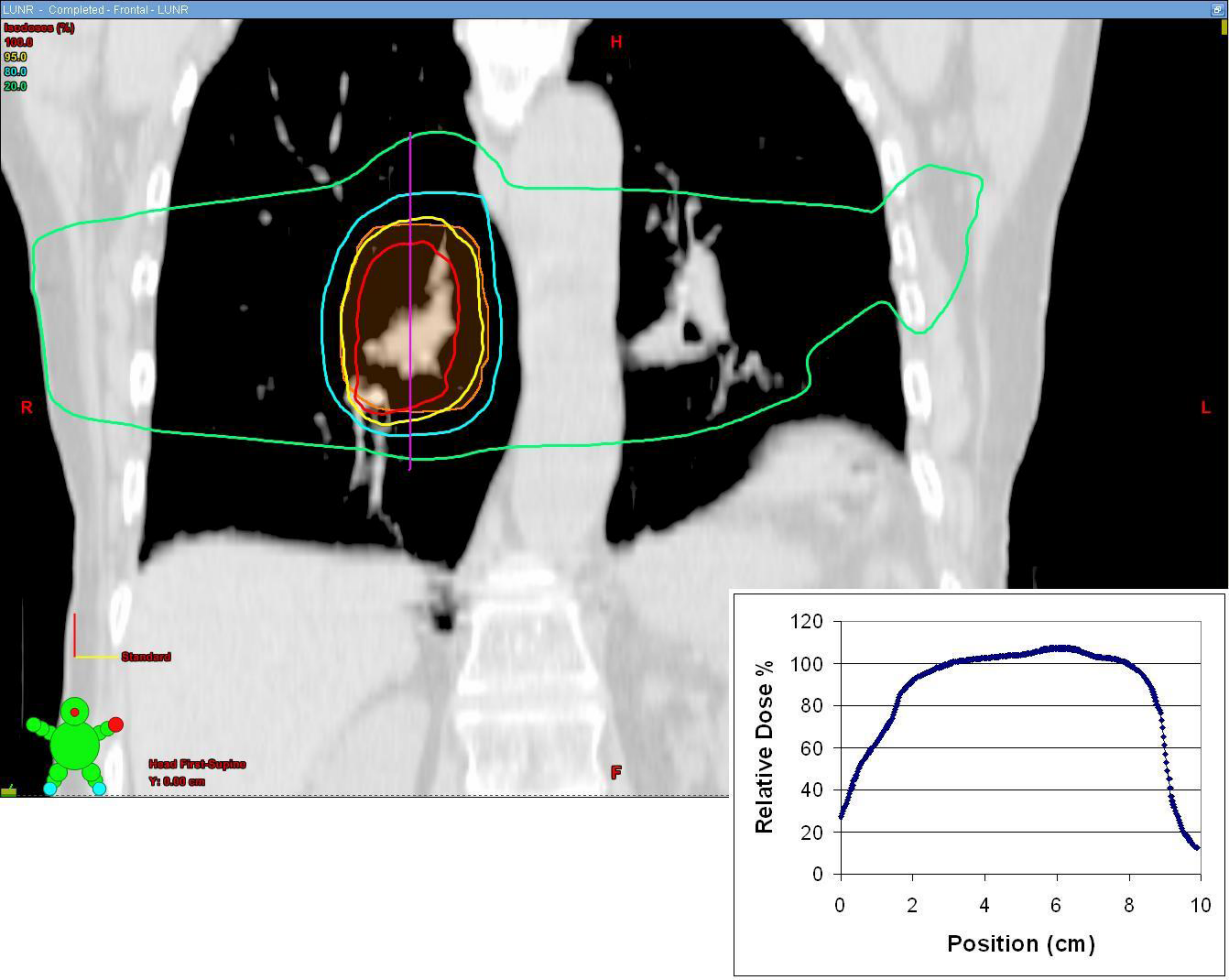
An image through the isocenter of the clinical three‐field treatment plan. The inset dose profile was used as the basis of the simulations of target motion in the case of the three‐field plan. The dose profile runs from superior to inferior through the center of the treatment volume along the vertical line indicated.

**Figure 10:**
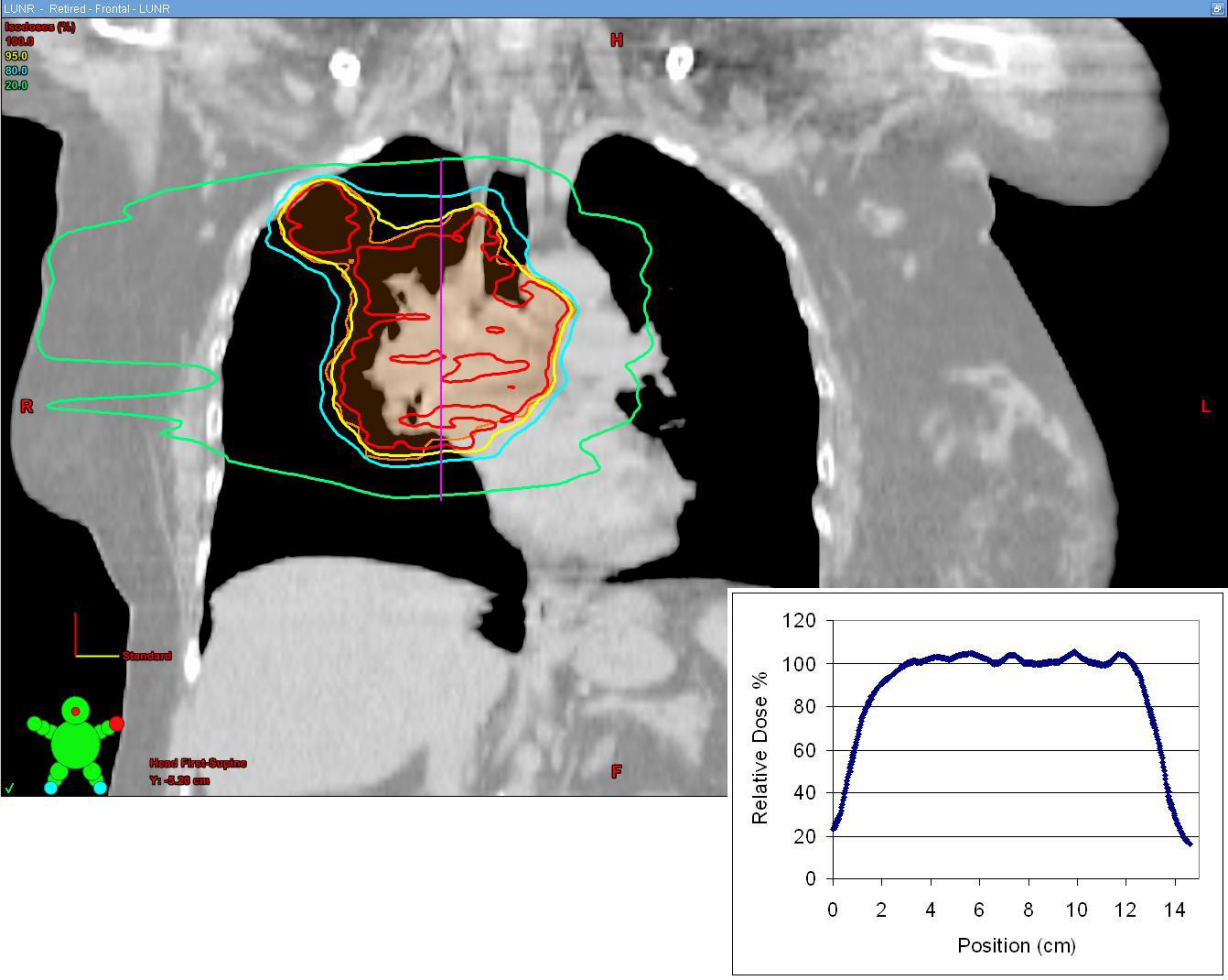
An image through the isocenter of the clinical arc treatment plan. This plan was comprised of two arcs. The inset dose profile was used as the basis of the simulations of target motion in the case of the arc plan. The dose profile runs from superior to inferior through the center of the treatment volume along the vertical line indicated.

**Figure 11:**
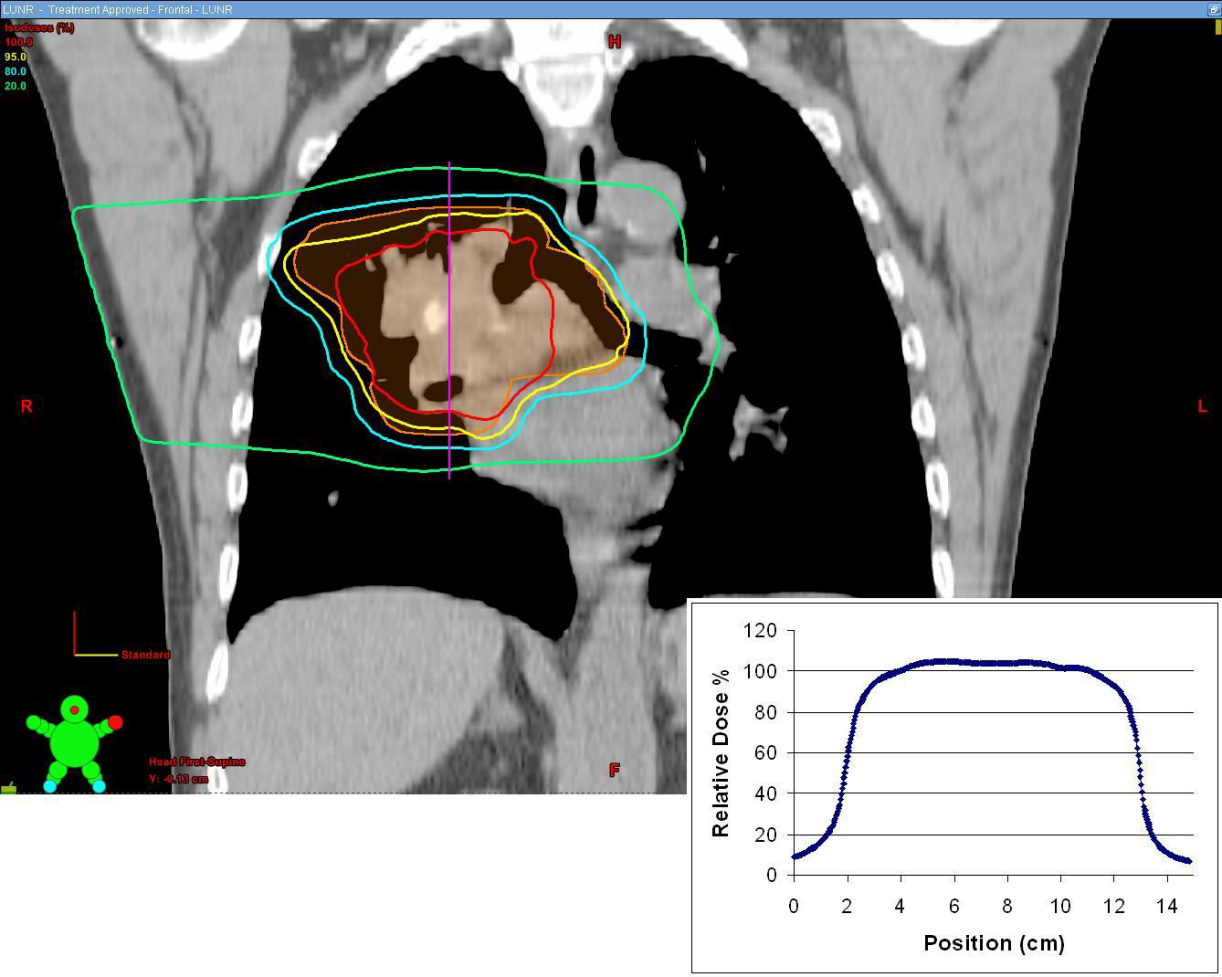
An image through the isocenter of the clinical four‐field treatment plan. The inset dose profile was used as the basis of the simulations of target motion in the case of the four‐field plan. The dose profile runs from superior to inferior through the center of the treatment volume along the vertical line indicated.

The analysis of the clinical treatment plans reveals that they are rather robust in the face of target motion. Figure 8 displays the loss of target $D_{95}$ versus the PDF standard deviation and also includes the phantom results of the 4.1 cm FWHM target for comparison. Images of the dose distributions and the associated dose profiles are displayed in Figs. 9 to 11.

The clinical plans lose very little $D_{95}$ for target motions which have a PDF standard deviation below 0.4 cm. It is only after the PDF standard deviation is larger than approximately 0.4 cm that the loss of target $D_{95}$ becomes appreciable. This highlights the effectiveness of selecting appropriate margins to compensate for target motion.

Since the size of the target plays an important role in the loss of $D_{95}$, it is instructive to look at the width of the dose profiles in these plans. The FWHM for the clinical three‐field, four‐field, and arc plans were measured at 8.5 cm, 11.1 cm, and 12.8 cm, respectively. The same trend that was identified with the study of target size can be seen in these three clinical plans, as well. However, the variation in dose gradients also plays a role in the clinical results. The results of the phantom study are generated with very steep dose gradients, resulting from a simple three‐field plan with overlapping beam edges. In practice, these phantom plan dose gradients will be as sharp as, or sharper, than what can be achieved in a clinical situation. As a result, the margin recommendations of the phantom plan can be taken as a worst case scenario, and can be safely applied to a clinical plan, which has equivalent or softer dose gradients.

## IV. DISCUSSION

### A. PDF gradients, standard deviations, and the convolution

When convolving a PDF with the static dose profile, there are two general aspects of the PDF which play a major role in the resulting blurred dose profile. They are the shape of the PDF and the range of the PDF.

**Figure 12:**
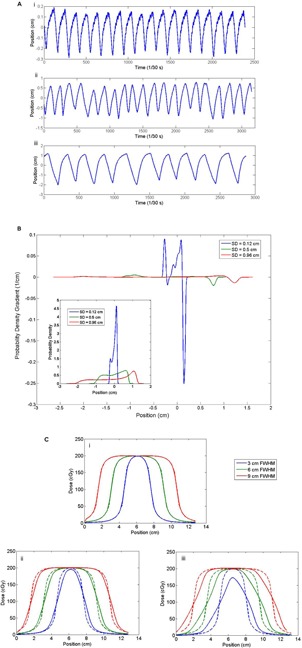
Three example breathing traces (a) from patients with a ‘regular’ breathing pattern: i) standard deviation of 0.12 cm; ii) standard deviation of 0.5 cm; iii) standard deviation of 0.96 cm. The derivatives (b) of the PDFs generated from each of the breathing traces with the original PDFs inset. While the shape of each PDF is similar (two mode shape), the range covered by each PDF differs drastically. The PDF asymmetry highlighted by the gradient contributes to profile asymmetry after convolution. The dose profiles (c) resulting from a convolution of each of the PDFs with dose profiles representing a range of target sizes. The dashed lines represent the original static profiles while the solid lines are the resulting blurred dose profiles: i) result of the convolution with the PDF with standard deviation of 0.12 cm; ii) result of the convolution with the PDF with standard deviation of 0.5 cm;. iii) result of the convolution with the PDF with standard deviation of 0.96 cm.

In Fig. 12(a) three breathing trace examples are presented. Each plot shows the properties of a trace categorized as a ‘regular’ breathing trace: little baseline drift, consistent breathing frequency, and consistent breathing amplitude. Figure 12(b) shows the PDFs generated from each of these breathing traces, with the area under each PDF being unity. It is immediately apparent that, although the patterns may all be considered ‘regular’, they are each fundamentally different, as described by the increase in standard deviation of each PDF. Furthermore, each of these traces demonstrates a different breathing frequency. Ultimately the differences in breathing frequency are accounted for by the PDF, which is a measure of the recorded positions over time. However, the breathing frequency can become an issue when it comes to meeting the sampling assumption inherent to the convolution model of target motion. In particular, if the breathing frequency is slow, it is more likely that sampling requirement will not be met. However, for typical dose rates (400 MU/min) and beam on times (∼10 s), the sampling requirement is sufficiently satisfied to allow for useful predictions from the convolution model. The implications of this assumption are discussed in more detail in the Results section E below.

Since each trace behaves in a regular fashion, each PDF shares the characteristic ‘two mode’ shape. However this two mode shape is spread out along the x‐axis differently among the three examples. The difference in spread of the PDF is ultimately tied to the amplitude of the motion, with increasing amplitude reflected in a larger standard deviation.

The effect of the two mode shape on the convolution is best assessed by looking at the gradients of these PDFs, as shown in Fig. 12(b). The asymmetry of these PDFs (compared to a standard Gaussian) is highlighted by plotting these gradients. The asymmetry results in an asymmetrical blurring of the static dose distribution after convolution. However, this effect is quite small in comparison to the effect of the PDF standard deviation.

As can be seen in Fig. 12(c), the resulting blurred dose profiles are strongly affected by the range of motion covered by the trace. This range of motion is characterized by the PDF standard deviation. As the standard deviation of the PDF increases, the resulting blurred dose profiles are strongly affected. Since this effect dominates the effect of the asymmetry, we can confidently assign margin selection recommendations based on the target size and PDF standard deviation.

### B. Margin selection

A wide variety of different breathing patterns were analyzed for their effect on the radiation dose distribution delivered by external‐beam radiation therapy. By including many traces collected from a variety of patients displaying both regular and irregular breathing patterns, it is anticipated that the data presented here are representative of a large population and, hence, the margin selection process may be applicable to any given patient.

Given a treatment planned on a static image of the patient with the target at its mean position and the renormalized patient breathing trace/PDF (generated from 4D CT data), the margin required to maintain target dose coverage can be determined from the data presented in this work. Because of the sharp penumbra at the edge of the dose profile, the phantom data can be seen to represent a worst case scenario. Therefore, the phantom data can be used to calculate the largest required margins based on a given breathing pattern.

First the loss of target coverage can be predicted using the results presented in Fig. 4 or Fig. 7, depending on the size of the target. In the case of the target $D_{95}$, the loss of coverage was well correlated with the PDF standard deviation and so each case can be read from the plot. Following the prediction of the coverage lost due to target motion, the PDF standard deviation can be used to reference the plot in Fig. 6. This will reveal how much additional target coverage is offered by each additional millimeter of beam width in the specific case of the patient. Finally, the additional margin is selected by determining the additional beam width required to restore the acceptable target coverage presented in the original treatment plan. Mathematically, the last step can be simply expressed as:

(5) $$ABW(mm)=RCL/RCR(mm^{-1})$$

where *ABW* is the Additional Beam Width, *RCL* is the Relative Coverage Lost (from Figs. 4 or 7), and *RCR* is the Relative Coverage Restored per additional mm of beam width (from Fig. 6).

For example, suppose a given patient presented with a breathing trace whose PDF has a standard deviation of 4 mm. After referencing Figs. 4 or 7, it is determined that the delivered $D_{95}$ is 96.7% of the originally planned value. Figure 6 shows that, for a PDF with 4 mm standard deviation, each additional mm of beam width restores 4% of the original $D_{95}$. Therefore, an additional 0.93 mm (≈1 mm) of beam width is required to compensate for the target motion in this case.

(6) $$\begin{array}{l} ABW=RCL/RCR(mm^{-1})\\ ABW=3.7\%/4\%(mm^{-1})\\ ABW=0.93mm\approx 1mm \end{array}$$

Although the breathing pattern PDF is required as input for the convolution model and not routinely gathered in the clinic, the information may be derived from 4D CT procedures. Generating the PDF can easily be automated through the use of scripts in a computational environment such as MATLAB. The simple program could be set up to run using only two inputs: 1) a breathing trace recording (as gathered during a 4D CT), and 2) the target motion amplitude (as measured on the 4D CT). From these inputs, the target coverage lost can be predicted and the required additional beam width can be determined. Table 3 summarizes the margins required to maintain the planned (static case) $D_{95}$ in the face of target motion in the worst case scenario represented by the phantom data. It is hoped that, by presenting the results in terms of a dose coverage metric commonly used in the clinic, these results would be useful for a clinician looking to select a motion compensating margin.

In comparison with the approach detailed by van Herk et al.,^(21)^ we can see that the margin recommendations presented here are similar for some situations. Although the van Herk formula makes no accounting for target size, we can compare the margin recommendations based on the study's “linear approximation of the random component for 95% dose coverage”. This is a simple formula which approximates the margin required to compensate for target motion as ‘0.7σ’ where σ is the standard deviation of the motion. These results are presented in Table 4. For small targets, the margins recommendations are similar, but for larger targets, the results computed in this study suggest less additional margin is required. The differences in margin recommendations likely arise due to differences in the approach to the problem. This study seeks the margin that will maintain $D_{95}$ for a specific patient, while van Herk and colleagues seek the margin that gives 95% coverage for 90% of patients.

**Table 3:** Summary of the recommended additional margin required (mm) to compensate for a loss of target $D_{95}$ due to target motion. As the full width at half max (FWHM) of the dose profiles increases, the required margins decrease. The final step in selecting a margin from this table requires the measurement of the PTV in the direction of motion of primary concern

		*PDF Standard Deviation (cm)*							
		*0.1*	*0.2*	*0.3*	*0.4*	*0.5*	*0.6*	*0.7*	*0.8*
*Dose Profile FWHM (cm)*	*2.6*	0.1 (mm)	0.4	0.8	1.4	2.3	3.6	5.4	7.9
	*3.1*	0	0.2	0.5	0.9	1.6	2.6	4.0	5.9
	*3.6*	0	0.2	0.4	0.8	1.4	2.2	3.4	5.1
	*4.1*	0	0.1	0.2	0.6	1.0	1.7	2.8	4.2
	*4.6*	0	0.1	0.2	0.5	0.9	1.5	2.4	3.7
	*5.1*	0	0	0.1	0.4	0.7	1.3	2.1	3.2
	*5.6*	0	0	0	0.2	0.5	1.0	1.8	2.9
	*6.1*	0	0	0	0.2	0.5	1.0	1.8	2.9
	*7.1*	0	0	0	0.1	0.4	0.9	1.6	2.6
	*8.1*	0	0	0	0.1	0.3	0.7	1.3	2.2
	*9.1*	0	0	0	0.1	0.3	0.7	1.3	2.1
	*10*	0	0	0	0	0.2	0.5	1.0	1.8

**Table 4:** A comparison of the margin recommendations presented in this work in comparison with the commonly used van Herk recommendations.^(21)^ For small targets, the recommended margins are similar, but for larger targets $D_{95}$ can be maintained with less additional margin than suggested by van Herk et al. Since van Herk and colleagues only claim accurate approximation of the formula used up to a SD of 0.5 cm, the values marked with an asterisk are noted as an extrapolation

*SD (cm)*	*0.1*	*0.2*	*0.3*	*0.4*	*0.5*	*0.6*	*0.7*	*0.8*
van Herk approximation	0.7 (mm)	1.4	2.1	2.8	3.5	$4.2^{*}$	$4.9^{*}$	$5.6^{*}$
2.6 cm FWHM	0.1	0.4	0.8	1.4	2.3	3.6	5.4	7.9
5.6 cm FWHM	0	0	0	0.2	0.5	1.0	1.8	2.9
10.1 cm FHWM	0	0	0	0	0.2	0.5	1.0	1.8

### C. Results of simulations on clinical plans

The result of the target motion simulation on the clinical plans reinforces the notion that the proper margin selection is an effective tool in compensating for target motion. The inherent clinical margins defining the PTV were left in place during these simulations. After including the modeled effect of target motion, the dose coverage still remained high for the majority of breathing patterns. It is only after the target motion becomes large on average (PDF standard deviation greater than 4 mm) that margins beyond what are typically applied should be considered in order to maintain target $D_{95}$.

Since the dose gradients play an important role in the loss of coverage, it is instructive to look at the width of the penumbra in the dose profiles near the beam edge of these plans. In the cases of the clinical three‐field and arc plans, the 80%–20% penumbra was measured at 14 mm (Figs. 9 and 10). In the case of the four‐field plan, the penumbra of the dose profile was 9 mm (Fig. 11). In each case, the dose gradient was less steep than in the phantom plan, which had a penumbra of 8 mm. The typical clinical plan (regardless of technique) will have dose gradients which are softer than those seen in the simple three‐field phantom plans. As a result, the margin recommendations generated from the phantom plans can be seen as a worst case scenario, and any plan with softer dose gradients will be sufficiently compensated for using the same margins.

### D. Example margin selection

Having determined recommended field margins shown in Table 3, it was instructive to reexamine the dynamic phantom dose profiles measured for a small target volume and extreme standard deviation (characteristic of a stereotactic radiosurgery case with large, irregular tumor motion). For this situation, the increase in field dimensions could compromise the dose to adjacent organs as seen in a treatment plan with adjusted margins. Clinically, a decision to use gated delivery instead of increased margin could be guided by the treatment plan with extended margins.

In order to validate the margin selection recommendations, an example case was tested. A treatment plan for a small target (2.6 cm FWHM dose profile) was first generated in Eclipse. A film measurement was taken as the treatment was delivered to the phantom while in dynamic mode. The phantom was programmed to replicate the motion of a recorded breathing trace with a large standard deviation (0.8 cm). A new plan was then generated by adding the appropriate margin from Table 3 to the small target. This new plan was then delivered to the phantom under the same motion conditions. The dose coverage offered by the new plan under motion was then compared to the original static plan in order to validate the use of Table 3 for margin selection recommendations.

**Figure 13:**
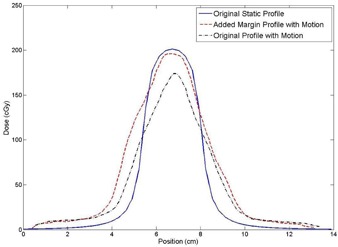
A plot of dose profiles demonstrating the margin recommendations provided in this work. The original static profile is taken from Eclipse, while the motion profiles were taken from dynamic film measurements. The original static plan loses dose coverage due to the target motion. When the margin recommendation from Table 3 is included in the plan, the target dose coverage is largely recovered when the treatment is delivered in the presence of the same target motion.

The results of this validation measurement are depicted in Fig. 13 and summarized in Table 5. The $D_{95}$ of the target PTV increases from 75.8% of the original $D_{95}$ without motion compensation to 95.4% with motion compensation. In this case, the additional margin would play an important role in ensuring an adequate dose is delivered to the treatment volume in the presence of target motion. The full $D_{95}$ was not entirely recovered because the particular breathing pattern used falls slightly below the trendline for dose recovered per millimeter additional beam width shown in Fig. 6 (point indicated).

**Table 5:** Summary of the results of the margin selection example to demonstrate use of the margin recommendations presented in this work. The $D_{95}$ of the PTV is largely recovered by including the margin recommended in Table 3

	*Original Static Profile (Eclipse)*	*Original Profile with Motion (film measurement)*	*Additional Margin Profile with Motion (film measurement)*
$D_{95}\;(\text{cGy})$	189.9	143.9	181.1
${\text{Relative D}}_{95}\;(\%)$	100	75.8	95.4

### E. Study limitations

The complexity of the problem of breathing motion in EBRT necessitates making some simplifying assumptions that ultimately limit the applicability of this study. The dose convolution model used to make predictions about the delivered dose in the presence of motion includes some specific assumptions. The model requires that the dose distribution delivered to the patient is constant throughout the treatment delivery, regardless of changes in patient anatomy.^(17)^ This assumption is never met in a strict sense; however the work of other authors has shown that the typical changes in anatomy due to breathing offer a negligible effect on the delivered dose distribution.^(22)^ Furthermore, the convolution model implicitly assumes that the input PDF is exactly representative of the target motion during treatment ‘beam on’. That is to say that the positions sampled by the target during ‘beam on’ must be equivalent to those sampled *a priori* to generate the PDF.^(23)^ If, for example, the treatment beam (or beam segment) is delivered with a high dose rate or with few MU, the amount of ‘beam on’ time may be insufficient for the target to complete its motion cycle and sample the traversed positions with the same relative intensity as measured *a priori*. This sampling requirement may not be met in a strict sense; however, some early analysis of the target position sampling required to meet this assumption shows that a high degree of correlation between the *a priori* PDF and the ‘beam on’ PDF was routinely achieved using typical dose rates (400 MU/min) and beam times (∼10 s) on the programmed breathing phantom. As a result, this assumption can usually be sufficiently satisfied as to make the model predictions useful. Newer 4D IGRT methods offer more reliable verification, which can be implemented to further ensure that this assumption is satisfied. The analysis presented here is only concerned with target motion in one dimension, which is not how target motion presents in general.^(5)^ Although the methods presented in this study will work to compensate for motion in any one arbitrary direction, more work is required to ensure the applicability of the method in two or three dimensions. In principle, we see no reason why this should not be possible. Finally, the treatment plans used to generate the margin predictions in this study were simple, open three‐field plans with overlapping beam edges in a phantom geometry. These simple plans were used in order to establish the trends one might expect to see in more complicated planning scenarios. In the case of modulated treatment techniques, such as IMRT or VMAT, the results presented here should be applied cautiously. Strictly speaking, the convolution model will only hold true if the motion that occurs during ‘beam on’ time is equivalent to the motion that was used to generate the PDF input. In the case of IMRT, this means that the target motion during the delivery of each beam segment needs to be very similar to the *a priori* motion. This may not be the case, especially for segment delivering very few MU. However, for a treatment spread over ~ 30 fractions, there will likely be enough additional averaging that the model will still make reasonably accurate predictions. This means that much additional care should be taken for hypofractionated schemes or techniques employing very high dose rates.

## V. CONCLUSIONS

The effect of target motion in the lung was analyzed for 502 unique patient breathing traces using a convolution model. The applicability of the model to intrafraction motion was verified for select breathing patterns with GAFCHROMIC film measurements in an anthropomorphic thorax phantom, set in both static and dynamic modes. A computational simulation revealed trends in target dose coverage loss due to intrafraction motion, which were strongly correlated to the standard deviation of the breathing trace. A linear trend in target dose coverage with changing field size was also identified. These trends can be used to determine the increase in field size required to compensate for target dose coverage loss due to intrafraction motion. A set of margin recommendations based on the target size and patient specific breathing pattern were presented.

## ACKNOWLEDGMENTS

The authors would like to thank Dr. Stewart Gaede of the London Regional Cancer Program for providing access to the breathing trace data.
